# The Effect of Upward Social Comparison on Academic Involution Among College Students: Serial Mediating Effects of Self-Esteem and Perceived Stress

**DOI:** 10.3390/bs15111515

**Published:** 2025-11-08

**Authors:** Ru Wen, Qingying Jin

**Affiliations:** Department of Psychology, School of Philosophy and Sociology, Jilin University, 2699 Qianjin Street, Changchun 130012, China; wenru0122@mails.jlu.edu.cn

**Keywords:** upward social comparison, self-esteem, perceived stress, academic involution

## Abstract

Academic involution in college students has a significant impact on their physical and mental health; however, its internal psychological mechanism remains unexplored. This study aimed to examine the relationship between upward social comparison and academic involution among college students and investigate the serial mediating role of self-esteem and perceived stress. A questionnaire survey, which included the Upward Social Comparison, Self-Esteem, Chinese Perceived Stress, and College Students’ Academic Involution Scales, was conducted with 730 college students via the random sampling method. The results revealed that all pairs of variables were significantly correlated. Furthermore, upward social comparison not only directly influenced college students’ academic involution, but also indirectly affected it through the separate mediating roles of self-esteem and perceived stress, as well as the serial mediating effect of both variables. This study enriches the theoretical mechanism of college students’ academic involution and offers empirical support for designing mental health education and intervention programs.

## 1. Introduction

As social competition has become increasingly intense in recent years, the term “involution” has emerged in Chinese society. It represents a phenomenon of excessive competition, which has been extensively applied in multiple areas and is especially serious in education (Y. Zhang et al., 2025). To obtain better academic evaluations or educational resources, people increase their investment and compete for small gains at great costs (X. Zhou et al., 2022). Furthermore, this phenomenon is extremely prominent among college students. At the individual level, college students’ academic involution refers to a cognitive strategy-oriented behavioral tendency to continue increasing academic investment despite the imbalance of payback in the context of excessive competition, accompanied by a certain emotional experience (J. Chen et al., 2024). With the devaluation of education and intensification of job market competition, campus pragmatism and utilitarianism are also increasing (Yu et al., 2022). College students’ performance is linked to various awards, opportunities, and resources; furthermore, it affects their future employment and development prospects (Y. Liu et al., 2022). Therefore, they do not hesitate to sacrifice efficiency, time, and resources (Yu et al., 2024), which makes involution particularly common and intense. However, the significance of academic involution extends beyond its prevalence to its profound impacts on both individuals and society. For individuals, excessive academic involution is closely associated with negative outcomes such as anxiety, depression, reduced motivation, diminished creativity, and academic burnout (Dou et al., 2022), serving as an important factor influencing college students’ psychology and behavior. At the macro level, intense involution disrupts the positive educational order and environment (S. Zhang et al., 2025), reflecting the latent contradictions in the allocation of educational resources and the structure of social opportunities, and is an important entry point for understanding the developmental challenges and psychological dilemmas of contemporary youth. It not only pertains to educational equity and social mobility, but also poses a challenge to the function of higher education in cultivating innovative talents.

Therefore, studying the antecedents of academic involution not only helps to enrich scholarly understanding of this emerging social phenomenon, but also provides a basis for developing educational interventions and policy measures, thereby enhancing college students’ learning and development while fostering a healthy educational environment. Previous research has demonstrated that upward social comparison is an important antecedent of academic involution (J. Chen et al., 2024). Moreover, upward social comparison represents the most common form of social comparison in daily life, particularly in competitive contexts (Gerber et al., 2018), which can reflect the typical psychological and behavioral situations faced by college students. Accordingly, using upward social comparison as the independent variable, the present study aims to construct a serial mediation model to explore how it affects academic introspection through the roles of self-esteem and perceived stress, in order to further explore and enrich the psychological mechanisms of college students’ academic involution.

### 1.1. Upward Social Comparison and Academic Involution

Upward social comparison refers to comparing oneself to those better than oneself (Jin et al., 2024). It is a significant predictor of college students’ academic involution (J. Chen et al., 2024). College years are a transition between school and society, when students often face great competition and uncertainty. According to the social comparison perspective, when people’s self-evaluation lacks an objective frame of reference, they tend to compare themselves with others to obtain information (Festinger, 1954; Suls et al., 2002). In general, individuals tend to engage in social comparison as soon as they are exposed to information about others (Festinger, 1954). In daily life, especially in competitive situations (Stapel & Koomen, 2005), people are more inclined to compare themselves with those better than them (Gerber et al., 2018), making upward social comparisons particularly prevalent. According to the assimilation effect of upward social comparisons (Collins, 1996), individuals will tend to behave similarly to the comparator, motivating individuals to enhance themselves and narrow the gap with their comparators (Garcia et al., 2013). However, in the broader social context of limited resources and opportunities, overly frequent upward social comparisons are often associated with negative outcomes, such as anxiety and stress (Tian et al., 2025). Research has shown that frequent upward social comparisons can have a significant impact on stress, negative emotions, and academic behaviors of students preparing for exams (Schlechter et al., 2025). In addition, they also affect students’ academic engagement (Qi et al., 2024), motivation (Wu & Shakibaei, 2025), and academic self-confidence (Hong et al., 2022). In the current context of social competition with limited resources and opportunities, when college students are continuously exposed to abundant information about others’ outstanding achievements, they may tend to continuously invest a great deal of energy in order to enhance their competitiveness, but ignore the trade-off between input and return, which ultimately exacerbates the occurrence of academic involution.

Based on the above, the following hypothesis H1 is proposed: Upward social comparison would positively predict college students’ academic involution.

### 1.2. Mediating Effect of Self-Esteem

Self-esteem is defined as an individual’s overall evaluation and emotional experience of their self-worth and self-competence (Rosenberg et al., 1995; P. Zhang et al., 2025). Numerous studies have found that upward social comparison impacts self-esteem. According to the contrast effect of social comparison, when individuals perceive that they are inferior to their comparators, their self-evaluation decreases, which can lead to various negative emotions, such as feelings of threat, emotional strain, and frustration (Collins, 1996; McComb et al., 2023). Upward social comparison decreases an individual’s self-evaluation and self-esteem and increases their risk of depression (Alfasi, 2019; Aubry et al., 2024). A study with 5215 students revealed that parents’ engagement in social comparison leads to adolescents engaging in upward social comparison, which in turn lowered their self-esteem (H. Liu et al., 2025). Moreover, upward social comparison in social networks contributes to a decline in adolescents’ self-esteem, which in turn leads to various problems, such as social anxiety (F. Yang et al., 2023), materialism (Hu et al., 2023), and cyberbullying (Gao et al., 2023).

In addition, self-esteem may also impact college students’ academic involution. The level of self-esteem affects how individuals evaluate the worth of their own opinions, and individuals with low self-esteem are more likely to be influenced by others when faced with self-choices and group choices, which can result in conformity (Enjaian et al., 2017). Low self-esteem leads to an increase in conformity behaviors such as alcoholism (Bainter & Ackerman, 2022). One study reported that when faced with inconsistency within the group, participants with low self-esteem were more inclined to conform to the way others were dressed next time (Chou et al., 2013). Therefore, individuals with lower self-esteem may be more susceptible to others’ and group influence in a collective and competitive university environment. In the current pervasive atmosphere of academic involution faced by Chinese university students (A. Liu et al., 2024), when observing that more “outstanding” peers continue to invest heavily in their studies, individuals with low self-esteem are more likely to be influenced by them and exhibit conformity tendencies, leading to greater investment in their own studies, thus increasing their tendency to engage in academic involution.

Therefore, hypothesis H2 was proposed: Self-esteem would mediate the relationship between upward social comparison and college students’ academic involution, such that upward social comparison would negatively relate to self-esteem, which in turn would negatively relate to academic involution.

### 1.3. Mediating Effect of Perceived Stress

Perceived stress refers to the level of stress experienced by an individual after subjective cognitive appraisal when faced with threatening environmental stimuli (Cohen et al., 1983). It is a combination of objective stressful events (stressors) and subjective cognitive evaluation of individuals. Upward social comparison is a significant risk factor for perceived stress and positively predicts its occurrence (Schlechter et al., 2025). On the one hand, Byron et al.’s (2010) research determined that information with social evaluation and comparison attributes is a psychological stressor. On the other hand, the contrast effect of upward social comparison triggers negative emotions, which lead individuals to be more sensitive to environmental stimuli and likely to perceive them as stressful, resulting in a higher level of perceived stress (Faelens et al., 2019). A study with 668 college students indicated that upward social comparison on online social networks positively predicted their perceived stress, which in turn increased mobile phone addiction (He et al., 2020).

In addition, perceived stress also impacted college students’ academic involution. A study on word frequency analysis and social semantic network analysis determined that stress was the most high-frequency emotional word associated with involution (Z. Chen, 2021). Another study, which defined individual psychological connotations of involution in Chinese society, reported that psychological stress is a key condition of involution, as suggested by interviews and questionnaires (W. Zhang et al., 2024). According to Lazarus and Folkman (1984), whether stress will affect an individual depends on their cognition and evaluation. Therefore, we introduced the concept of perceived stress and considered it a factor that influenced involution. According to the Transactional Model of Stress and Coping (Lazarus & Folkman, 1984), perceived stress activates coping mechanisms that prompt individuals to adopt appropriate strategies in order to restore psychological balance. Coping strategies are generally classified into two types: Problem-Focused Coping (dealing with the problem directly in response to the stressor) and Emotion-Focused Coping (regulating one’s own emotional responses). In a competitive situation during college, peer excellence information from upward social comparison often makes individuals feel a gap with others (Xu & Li, 2024). Coupled with the limited nature of competing resources, this sense of disparity is exacerbated, triggering higher levels of perceived stress. To cope with this stress, and driven by problem-focused coping strategies, individuals tend to increase their sustained academic engagement in an effort to narrow the gap with others, thereby exhibiting a stronger tendency toward academic involution.

Hence, hypothesis H3 was proposed: Perceived stress would mediate the relationship between upward social comparison and college students’ academic involution, such that upward social comparison would positively relate to stress, which in turn would positively relate to academic involution.

### 1.4. Serial Mediating Effect of Self-Esteem and Perceived Stress

Self-esteem has a significant effect on perceived stress. According to the transactional model of stress and coping, the perception of stress involves two processes: primary (assessing the current stimulus event and how much it affects oneself) and secondary appraisal (assessing the personal resources that can be used to cope with stress; Lazarus & Folkman, 1984). And self-esteem can enhance the perception of available resources and perceived validity, or serve as a resource for secondary assessment (Juth et al., 2008), thus having a significant impact on the perception of stress (Wright et al., 2023). At higher levels of self-esteem, people are better able to cope with stimulus events and mitigate negative impacts, thus perceiving lower levels of stress (Byrne et al., 2007; Ma et al., 2024; Tang et al., 2024). Conversely, as self-esteem decreases, individuals perceive stress more sensitively. A study with 14,000 students found that self-esteem was a significant indicator of perceived stress as well as physical and mental health (Stupnisky et al., 2013). Another study in the pandemic context suggested that self-esteem reduced perceived stress, as it increased perceived social support and reduced anxiety (H. Chen et al., 2021). Therefore, in a highly competitive and uncertain environment, college students are prone to frequent and intense upward social comparison, which may lower self-esteem, thus causing them to perceive higher levels of stress, and ultimately increasing their tendency toward academic involution.

Therefore, this study proposed hypothesis H4: self-esteem and perceived stress play serial mediating roles in the relationship between upward social comparison and college students’ learning involution.

**Figure 1:**
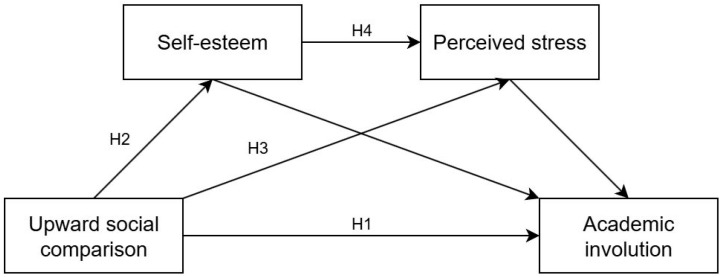
Serial mediation hypothesis model.

In summary, this study introduced self-esteem and perceived stress to construct a serial mediation model (Figure 1) for the first time to explore the interrelationships among these four variables and potential psychological mechanism of how upward social comparison affects college students’ academic involution.

## 2. Materials and Methods

### 2.1. Participants

This cross-sectional study employed a questionnaire survey, which was administered online using the survey platform “Wenjuanxing” (wjx.cn). The questionnaire was distributed in August 2024 and students from multiple universities across China were recruited. Data were collected in one round, and all participants took part voluntarily. The study’s purpose and confidentiality policy were explained to the participants in detail before they completed the questionnaire. All participants provided informed consent. Of the 863 questionnaires distributed, 730 valid questionnaires were obtained after invalid questionnaires that failed the detection questions (i.e., “Please select the non-compliant option for question 15”; “Please select the occasional option for question 41”) or had consistent answers were excluded (effective rate of 84.59%). Participants’ mean age was 20.37 years (SD = 1.939), and 255 were men (34.9%) and 475 were women (65.1%). Furthermore, the majority of participants were undergraduates (91.0%), ranging from first- to fourth-year students, and 9.0% were postgraduates.

### 2.2. Measures

#### 2.2.1. Upward Social Comparison Scale

This study used the upward social comparison subscale of the Iowa-Netherlands Comparison Orientation Measure developed by Gibbons and Buunk (1999) and revised by Bai et al. (2013). The scale comprises six items scored on a 5-point scale. The higher the total score, the higher the degree of upward social comparison. In this study, Cronbach’s alpha coefficient was 0.890.

#### 2.2.2. Self-Esteem Scale

Rosenberg’s Self-Esteem Scale (Rosenberg, 1965) translated by X. Wang et al. (1999) was used. It comprised 10 items scored on a 5-point scale, and Items 3, 5, 8, 9, and 10 were reverse scored. Higher scores reflected greater self-esteem. In this study, Cronbach’s alpha coefficient was 0.870.

#### 2.2.3. Chinese Perceived Stress Scale

The Chinese Perceived Stress Scale developed by Cohen et al. (1983)and translated and revised by T. Yang and Huang (2003) was used. It comprised 14 items and two dimensions: tension (assesses individuals’ emotional states under the influence of stressors) and loss of control (evaluates individuals’ ability to cope with stressful events). Each item was rated on a 5-point scale, and Items 4, 5, 6, 7, 9, 10, and 13 were reverse scored. Higher scores reflected greater perceived stress. In this study, Cronbach’s alpha coefficient was 0.841.

#### 2.2.4. College Students’ Academic Involution Scale

The College Students’ Academic Involution Scale developed by J. Chen et al. (2023) was used. It comprised 24 items scored on a 5-point scale and was divided into five dimensions: emotional arousal (assesses students’ emotional experiences during academic involution), behavior recognition (assesses various involution-related academic behaviors), state monitoring (evaluates students’ cognitive awareness of academic involution), expected goals (captures students’ goal orientation during involution), and return trade-offs (assesses students’ consideration of the balance between academic effort and perceived rewards). Higher scores indicated a higher degree of individual academic involution. In this study, Cronbach’s alpha coefficient was 0.920.

### 2.3. Data Analysis

SPSS version 20.0 and PROCESS version 3.5 were used for data analysis. First, we standardized the data of the variables. Harman’s single-factor analysis was used to assess the common method bias. Descriptive statistics and Pearson’s correlation analyses were performed on the main variables. Finally, the serial mediation effect was analyzed via PROCESS Model 6 and assessed via the bootstrap method with 5000 resamplings (95% confidence interval).

## 3. Result

### 3.1. Common Method Bias

The use of self-report measures in data collection may have introduced common method bias. To reduce potential bias, certain procedural controls were performed during the survey, such as anonymous distribution of questionnaires and use of reverse scoring (H. Zhou & Long, 2004). Harman’s single-factor test was used to analyze common method bias, and the results revealed 10 factors with eigenvalues greater than 1. The variation explained by the first factor was 27.230%—less than 40%—which indicated no significant common method bias (H. Zhou & Long, 2004).

### 3.2. Descriptive Statistics and Correlation Analysis

Descriptive statistics and correlation analyses were performed, and the results are presented in Table 1. Upward social comparison was significantly negatively correlated with self-esteem (*r* = −0.207, *p* < 0.01) and positively correlated with perceived stress (*r* = 0.355, *p* < 0.01) and academic involution (*r* = 0.481, *p* < 0.01). Furthermore, self-esteem was significantly negatively correlated with perceived stress (*r* = −0.684, *p* < 0.01) and academic involution (*r* = −0.497, *p* < 0.01). Perceived stress was significantly positively correlated with academic involution (*r* = 0.594, *p* < 0.01).

### 3.3. Mediation Effect Test

SPSS version 20.0 and PROCESS macro Model 6 were used to analyze the serial mediating role of self-esteem and perceived stress in the effect of upward social comparison on college students’ academic involution. Bootstrap method with 5000 resamplings (95% confidence interval) was used for assessment. Gender, age, and grade were used as control variables to exclude their effects. Table 2 presents the results. Upward social comparison significantly negatively predicted self-esteem (β = −0.208, *p* < 0.001) and significantly positively predicted perceived stress (β = 0.206, *p* < 0.001) and academic involution (β = 0.311, *p* < 0.001). Self-esteem significantly negatively predicted perceived stress (β = −0.640, *p* < 0.001) and academic involution (β = −0.180, *p* < 0.001). Furthermore, perceived stress significantly predicted academic involution (β = 0.366, *p* < 0.001).

Table 3 presents the results of the mediating pathway. The mediating effects of self-esteem and perceived stress between college students’ upward social comparison and academic involution were significant (95% confidence interval [CI] does not include 0), with a total mediating effect value of 0.161, which accounted for 34.11% of the total effect. Among them, the mediating effect sizes of pathways 1 (upward social comparison → self-esteem → academic involution), 2 (upward social comparison → perceived stress → academic involution), and 3 (upward social comparison → self-esteem → perceived stress → academic involution) were 0.037 (95% CI = [0.018, 0.062]), 0.075 (95% CI = [0.051, 0.102]), and 0.049 (95% CI = [0.029, 0.070]), which accounted for 7.84%,15.89%, and 10.38% of the total effect, respectively. No pathways had a 95%CI that included zero, which indicated that all three indirect effects were statistically significant. At this point, we established a partial mediation serial model.

**Figure 2:**
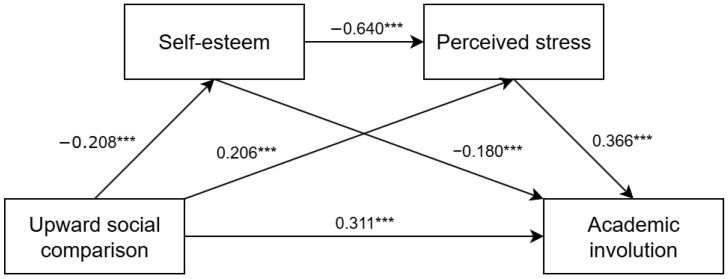
Path analysis of the serial mediation model. Notes: This figure illustrates the results of the path analysis that assessed the serial mediation model. All path coefficients were standardized. *** indicates statistical significance at *p* < 0.001.

A serial mediation model diagram of the impact of upward social comparison on college students’ academic involution is presented in Figure 2.

## 4. Discussion

### 4.1. Impact of Upward Social Comparison on Academic Involution

The results indicate that upward social comparison positively predicted college students’ academic involution. Hence, upward social comparison is a key factor in contemporary college students’ academic involution, and H1 was confirmed. This result is consistent with that of a previous study (J. Chen et al., 2024). During college years, individuals often need to engage in competition for grades, resources, or opportunities (F. Wang et al., 2024). However, unlike high school, college lacks clear objective evaluation criteria. Therefore, college students often engage in social comparison to obtain information about their relative status, abilities, and performance, which provides a basis for relevant judgments and decisions, thereby reducing uncertainty (Stapel & Koomen, 2005). Owing to the spontaneous tendency to compare with the best (Gerber et al., 2018) and practical need to outperform competitors to gain an advantage for future study and work, college students engage in a great deal of upward social comparisons. According to the assimilation effect of upward social comparison (Collins, 2000), individuals develop a spontaneous “upward drive,” which motivates college students to adopt various strategies to enhance their academic engagement, thereby improving themselves and narrowing the gap with their comparison targets. However, in the current context of limited social resources and opportunities, excessive and frequent upward social comparisons may lead students to continuously increase their academic investment to catch up with others, neglecting the cost–benefit balance of their investment, and ultimately contributing to academic involution. In addition, from the perspective of relative deprivation theory, perceived disadvantage in comparison with others or groups can trigger relative deprivation (Zhao et al., 2024); this feeling of resource deprivation will stimulate a strong sense of competition, prompt college students to continuously increase their investment to improve their competitiveness, and ultimately increase academic involution (A. Liu et al., 2024).

### 4.2. Role of Self-Esteem as a Mediator

The results show that self-esteem mediates the relationship between upward social comparison and college students’ academic involution. Hence, H2 was confirmed. First, upward social comparison negatively predicted self-esteem, which suggested that frequent upward social comparison threatened an individual’s self-esteem, supporting previous results (Aubry et al., 2024). According to the social comparison theory, a significant contrast effect of upward social comparison will lead to a decrease in individual self-evaluation and an increase in negative emotions (McComb et al., 2023). Therefore, after being exposed to substantial information about the excellence of others, especially in a competitive environment, college students are more likely to focus on the disparity between themselves and their comparison targets and feel a negative sense of “not as good as others.” This in turn fosters doubt and devaluation of their own abilities and worth and ultimately results in a decline in self-esteem. Second, self-esteem negatively predicted college students’ academic involution, which indicated that those exhibiting low self-esteem were more prone to undergo involution in college studies than individuals with high self-esteem. Individuals with lower self-esteem evaluate themselves more negatively and are more likely to doubt the correctness of their own attitudes and behaviors, and thus tend to conform to groups (Enjaian et al., 2017). Therefore, these students are more likely to imitate irrationally in a strong academic involutional atmosphere and increase involution tendency. Especially after making an upward social comparison, others’ success gives individuals a type of reinforcement, which can further enhance their conformity tendency (Field et al., 2024). In addition, from a cognitive perspective, unlike individuals with high self-esteem, individuals with low self-esteem have more depersonalized thinking and are less likely to present their own unique opinions about real-world situations (Cen & Zhen, 2004). Therefore, in a fierce competitive situation, college students with high self-esteem are more likely to interpret information by relating it to the context of things and their own characteristics, engage in purposeful and rhythmic learning, and reduce the involutional behavior of blind emulation.

### 4.3. Role of Perceived Stress as a Mediator

The results suggested that perceived stress mediated the relationship between upward social comparison and college students’ academic involution. Hence, H3 was confirmed. First, upward social comparisons positively predicted perceived stress, which suggested that frequent upward social comparisons lead to greater perceived stress, supporting earlier results (He et al., 2020). On the one hand, upward social comparison provided substantial information about the excellence of peers, which became a type of stressor and influenced college students’ perceived level of stress (Byron et al., 2010). On the other hand, Feinstein et al.’s (2013) study revealed that individuals were more likely to magnify their own shortcomings and evaluate themselves more negatively in the process of upward social comparison, which led to higher levels of perceived stress. Second, perceived stress positively predicted college students’ academic involution, which indicated that individuals were more likely to involute when they perceived a high level of stress, which is consistent with the results of previous studies (Yan et al., 2022). In a highly competitive environment with limited resources and opportunities, the abundance of information about the superior performance of others may lead to significant pressure on college students (Xu & Li, 2024), and in order to cope with this pressure and reduce the gap with others, individuals will continue to increase their academic commitment, ultimately resulting in academic involution. In addition, from the emotional perspective, emotions significantly impact human behavior, and excessive stress can lead to irrational coping and extreme behaviors (Yan et al., 2022). In college, students need to compete fiercely for resources and opportunities (F. Wang et al., 2024), and the intense pressure forces them to invest extra effort to outperform their competitors, even if their decisions and behaviors are irrational (Yi et al., 2022). Therefore, perceived stress is an important part of college students’ academic involution.

### 4.4. Serial Mediating Role of Self-Esteem and Perceived Stress

These results indicated that self-esteem and perceived stress played Serial mediating roles in the relationship between upward social comparison and college students’ academic involution. Hence, H4 was confirmed. Self-esteem affected the level of perceived stress, and even in the same situation, the stress experienced by individuals could vary with the level of self-esteem (S. Wang et al., 2023). According to Lazarus and Folkman’s (1984) transactional model of stress and coping, an individual’s assessment of resources (secondary appraisal) influences their perception of stress. And self-esteem can enhance the perception of available resources and the validity of perceptions, thus having an important impact on stress perception (Wright et al., 2023). When individuals have higher levels of self-esteem, they are more likely to evaluate stimulating situations as challenges rather than threats, thus perceiving less pressure (Meijen et al., 2020). Moreover, even in the face of setbacks and threats, self-esteem can serve as a psychological resource for secondary assessments (Juth et al., 2008) that helps individuals cope, resulting in lower levels of perceived stress, less negative emotions (Kupcewicz et al., 2024), and lower physiological cortisol levels (Wojtyna et al., 2024). However, in a highly competitive and uncertain environment, college students who frequently engage in upward social comparisons are more likely to feel a gap with others, which is often linked to negative self-evaluation and lower self-esteem. As self-esteem levels decrease, individuals become more sensitive to external stimuli and have progressively fewer positive psychological resources, making them more likely to perceive high levels of stress and ultimately increasing the tendency to academic involution.

### 4.5. Research Implications

Involution is a common phenomenon in education which significantly affects students’ physical and mental health. Many previous studies have discussed involution at the macro level in areas, such as politics, economy, and culture, while limited empirical studies are based on quantification (Dou et al., 2022). This study adopts a micro-level perspective to empirically investigate academic involution among college students from a psychological standpoint, thereby contributing to theory development at the individual level. Evidence suggests that upward social comparison is a critical factor influencing academic involution, yet the underlying mechanisms remain insufficiently understood. Drawing on social comparison theory, the present study introduces self-esteem and perceived stress as mediating variables, revealing how upward social comparison influences academic behaviors through multiple psychological pathways and elucidating the development of academic involution. which not only enriches the theoretical framework of academic involution but also extends the applicability of social comparison theory. Moreover, it complements existing research on self-esteem and perceived stress, highlighting their significance in academic contexts, thereby broadening the scope of inquiry and providing a theoretical foundation for future studies.

In addition, this study provides several practical implications at both the individual and macro levels. At the individual level, it provides a theoretical basis for developing intervention measures for college students’ academic involution. For example, from the perspective of perceived stress, cognitive behavioral therapy (González-Valero et al., 2019), mindfulness-based stress reduction therapy (Ritvo et al., 2021), and acceptance commitment therapy (Binder et al., 2024) can be used to help college students rationally view environmental stimuli and reduce their level of perceived stress to improve academic involution and alleviate the related negative emotions, cultivate a positive and healthy attitude toward life and study, and effectively promote the development of physical and mental health. At the macro level, this study suggests that universities, policymakers, and society should actively guide young people to objectively evaluate information presented by others, strive to reduce the negative impact of upward social comparison, and expand its positive driving role. In addition, policymakers should pay attention to educational equity and the fair distribution of resources to promote rational academic competition. Furthermore, this study highlights the importance of positive self in college students’ growth and development, which offers guidance for future work related to mental health education in colleges and universities.

### 4.6. Limitations and Prospects

Despite its contributions, the present study has limitations that warrant consideration in future research. First, the cross-sectional design of the present study somewhat limits the ability to draw clear causal inferences among the variables. Future research could incorporate experimental or longitudinal designs to not only test causal pathways but also examine how these psychological and behavioral variables evolve over time, thereby providing more robust theoretical evidence. Second, there remains room for improvement in the reliability and validity of the scales used in this study, and future research could consider employing more rigorous methods to further assess model fit. Furthermore, the mechanisms between upward social comparison and college students’ academic involution are complex, and this study focused only on the roles of self-esteem and perceived stress. Other variables can be introduced in future studies to consider the effects of more complex mechanisms or multiple factors. In addition, this study limited the target group to college students and explored the involution mechanism in the academic field, which limits the generalizability of the results. Future studies can expand to other fields and extend the target group to broaden the field and scope of research.

## 5. Conclusions

The results of this study suggested that upward social comparison positively predicted college students’ academic involution. Furthermore, this relationship was serially mediated by self-esteem and perceived stress. Our results enrich the theoretical mechanism of college students’ academic involution and offer empirical support for designing mental health education and intervention programs in colleges.

## Figures and Tables

**Table 1:** Descriptive Statistics and Correlation Analysis of the Variables.

Variables	M	SD	1	2	3	4	5	6	7
1. Gender	-	-	1						
2. Age	20.370	1.939	−0.086 *	1					
3. Grade	2.500	1.271	−0.135 **	0.814 **	1				
4. USC	3.464	0.890	−0.048	0.119 **	0.086 *	1			
5. SE	3.588	0.660	0.050	−0.013	−0.027	−0.207 **	1		
6. PS	2.908	0.577	−0.038	0.020	0.060	0.335 **	−0.684 **	1	
7. AI	3.033	0.679	−0.102 **	0.111 **	0.093 *	0.481 **	−0.497 **	0.594 **	1

Notes: Gender is a dummy variable, female = 0, male = 1; * *p* < 0.05; ** *p* < 0.01. Abbreviations: USC, upward social comparison; SE, self-esteem; PS, perceived stress; AI, academic involution.

**Table 2:** Regression analysis of the variable relationships in the serial mediation model.

Variables	SE		PS		AI	
	β	t	β	t	β	t
Gender	0.038	1.038	0.009	0.355	−0.062	−2.268 *
Age	0.054	0.866	−0.098	−2.165 *	0.098	2.090 *
Grade	−0.048	−0.765	0.106	2.342 *	−0.049	−1.031
USC	−0.208	−5.676 ***	0.206	7.674 ***	0.311	10.703 ***
SE			−0.640	−24.046 ***	−0.180	−4.841 ***
PS					0.366	9.438 ***
R	0.213		0.715		0.695	
R^2^	0.046		0.511		0.469	
F	8.640 ***		150.989 ***		106.435 ***	

Notes: Variables in the model were standardized and incorporated into the regression equation. * *p* < 0.05; *** *p* < 0.001. Abbreviations: β, Standardized coefficient; SE, standard error; USC, upward social comparison; SE, self-esteem; PS, perceived stress; AI, academic involution.

**Table 3:** Mediation effect analysis of the model.

Path	Effect	Boot SE	Boot 95% CI Lower Limit	Boot 95% CI Upper Limit	Relative Mediation Effect
USC→SE→AI	0.037	0.011	0.018	0.062	7.84%
USC→PS→AI	0.075	0.013	0.051	0.102	15.89%
USC→SE→PS→AI	0.049	0.011	0.029	0.070	10.38%
Total indirect effect	0.161	0.020	0.121	0.201	34.11%
Direct effect	0.311	0.029	0.254	0.368	65.89%
Total effect	0.472	0.033	0.408	0.536	100.00%

Abbreviations: USC, upward social comparison; SE, self-esteem; PS, perceived stress; AI, academic involution.

## Data Availability

The data from the present study are available from the corresponding author upon reasonable request.

## Abbreviations

The following abbreviations are used in this manuscript:

CI	Confidence interval
USC	Upward social comparison
SE	Self-esteem
PS	Perceived stress
AI	Academic involution
